# Secret Remedies and Proprietary Products.—II

**Published:** 1908-02-08

**Authors:** 


### SECRET REMEDIES AND PROPRIETARY PRODUCTS.—II.

When a quack finds it difficult to make a living in one place, he goes to another, and there having exploited his quackery until such time as his fraudulent practices are exposed, he sets out in search of fresh victims elsewhere. As time goes on and country after country closes its doors on quackery, the whole tribe of nostrum mongers is concentrated on the gradually diminishing area that remains open; and so it happens that in Great Britain at the present time, professional advertisers, the press, street hoardings, the Post-office, the Inland Revenue, and everything else that can serve the purpose of dealers in secret frauds are being subsidised by capital accumulated as the result of dishonest trading. Banned from the Continent, repudiated by some of the British possessions, and woefully exposed in America, the nostrum is placed under no restriction in this country. Since the new Commerce Act went into force in Australia, and certain restrictions were placed upon the importation of secret remedies into Commonwealth ports, and the exposure of fraud and the passing of the Pure Food Act in the United States, Great Britain has been the centre of unprecedented activity on the part of the quacks. Never before in the history of fraud has money been spent so lavishly, much of it, of course, by way of "hush money."

It is calculated that in England at the present time over 10,000 secret remedies are advertised to the public, while 10,000 varieties of branded products are advertised to the medical profession. With the exception that the Pharmacy Act, 1868, requires that medicines containing certain poisons must be so labelled and only sold by registered chemists, the law, in actual experience, imposes no restriction whatever on the sale of these products. However noxious the preparations may be, however fraudulent, whatever claims are made for them, whatever may be the misrepresentations made as to their compositions, the proprietors of these compounds are unchecked in their practices. It seems strange, however, that the Merchandise Marks Act is never enforced against a manufacturer who tells a physician that his branded product is a definite chemical compound of such and such a character, while it may really be an ordinary mixture of a totally different nature from that represented. It is equally difficult to understand why criminal proceedings are not instituted against the hundreds of proprietors of secret remedies, who deliberately obtain money by false pretences.

A short time ago an unsuccessful attempt was made by the Health Committee of the Corporation of Liverpool to repress the sale of a certain class of nostrum by bringing into operation Section 27 of the Sale of Food and Drugs Act, 1875. Although it is an offence under Section 6 of this Act "to sell to the prejudice of the purchaser any article of food or any drug which is not of the nature, substance, and quality of the article demanded by such purchaser," there is a special exemption from the operation of this section in cases "where the drug or food is a proprietary medicine, or is the subject of a patent in force, and is supplied in the state required by the specification of the patent." The unfortunate effect of this exemption on the public health and the public pocket will be shown in due course; in the meantime, it will suffice to say that the Liverpool Corporation was unable to proceed under this Section, and therefore instituted proceedings against the retailer of an advertised tonic, under the last paragraph of Section 27 of the same Act, which provides that "every person who shall wilfully give a label with any article sold by him which shall falsely describe the article sold, shall be guilty of an offence under this Act, and be liable to a penalty not exceeding twenty pounds." According to the evidence of the public analyst for the city of Liverpool, the article which was alleged to be falsely described as a tonic and digestive medicine, was innocent of digestive properties. As the composition of the "remedy" was not known to the retailer, it was hardly possible to prove that he knew it to be falsely described, and the summons was withdrawn, the defendant being allowed costs. Although this prosecution was unsuccessful in so far as it failed in its immediate intention, it was productive of at least one useful result. It drew the attention of the retailer to the fact of which he appears to have been ignorant, that if he sells a nostrum, knowing that there is attached to it "wilfully" a label, which "falsely describes" the article, he is liable to the infliction of a penalty of £20. In many cases it is so very obvious that secret medicines are falsely described that the retailer of such products would find it extremely difficult to maintain the defence that he was acting without guilty knowledge. A further purpose served by this prosecution was the reminder that although the Sale of Food and Drugs Act does provide means for checking misdescription of proprietary articles, the remedy is never enforced against the true culprits, the actual manufacturers of fraudulent nostrums.

It is well, at the outset, to draw attention to the fact that there already exists machinery for dealing, at any rate with a very large proportion of "patent medicines," as will probably be pointed out when Parliament is asked, as it will be at an early moment, for legislation for the suppression of quackery. As this machinery is seldom, if ever, put in motion in respect of fraudulently advertised remedies, it might just as well be non-existent.

